# Assessment of knowledge, attitude, and practice on early childhood caries among dental undergraduates and residents in China

**DOI:** 10.1186/s12909-024-05188-6

**Published:** 2024-03-04

**Authors:** Jingjing Yu, Guangtai Song, Jian Yu

**Affiliations:** Department of Paediatric Dentistry, State Key Laboratory of Oral & Maxillofacial Reconstruction and Regeneration, Key Laboratory of Oral Biomedicine Ministry of Education, Hubei Key Laboratory of Stomatology, School & Hospital of Stomatology, Wuhan University, Wuhan, China

**Keywords:** Child, Dental, Early childhood caries, Education, Oral health

## Abstract

**Background:**

Early childhood caries (ECC) causes severe, widespread oral health issues in children. Dental undergraduates and residents are expected to have a solid understanding of ECC for children’s oral health promotion. This study aimed to evaluate the knowledge, attitude, and clinical practice on ECC among dental undergraduates and residents in China.

**Methods:**

A 23-item electronic questionnaire was distributed to 598 dental undergraduates (4th- and 5th-year undergraduates) and residents (1st-, 2nd-, and 3rd-year residents) at the School of Stomatology, Wuhan University, China (in April–May 2023). SPSS Statistics was used to analyze the data using the Chi-square test at a significance level of 0.05.

**Results:**

A total of 422 questionnaires were completed by participants (recovery rate: 70.6%) from various academic levels. Around 77.3% of participants had heard of ECC (mainly from textbooks), and only 27.5% considered themselves familiar with it. Residents (79.8%) had higher risk awareness of ECC on children’s overall health than undergraduates (58.3%) (*p* < 0.05), but only 54.0% of participants correctly defined ECC. Most participants had a positive understanding of ECC’s pathogenic factors and preventive measures, including feeding patterns (71.6%), fluoride application (93.4%), and teeth cleaning (93.1%). Furthermore, only 50.2% of participants encountered ECC cases in clinic.

**Conclusions:**

Despite having a suboptimal level of ECC-related knowledge and practice, dental undergraduates and residents in China demonstrated a more positive attitude towards its etiology-based prevention. Strengthening ECC education, guidance, and practice may enable them to gain a better understanding of ECC learning, which would benefit children’s oral health.

## Introduction

Early childhood caries (ECC), a chronic infectious disease of childhood that leads to significant oral health problems, is defined as the “presence of one or more decayed, missing, or filled tooth surfaces in any primary tooth in a child aged 71 months or younger” [1]. Several factors contribute to the development and progression of ECC. The primary focus of investigating the etiology of ECC lies in understanding the caries core microbiome, oral micro-ecological imbalance, and their interactions with host genetic factors [2]. These aspects facilitate ECC’s pathogenesis study and serve as the theoretical foundation for developing ecological treatment and prevention. The high consumption of sugars is a significant risk factor related to the microbial etiology of ECC. The role of diet in the etiopathogenesis of ECC is well established [3]. Although there are clear benefits to breastfeeding in a child’s first year of life, use of breastfeeding and baby bottles after the age of one year, particularly if nocturnal and/or frequent, is associated with ECC [4].

ECC has a high prevalence and incidence, and the distribution is uneven. It was reported in 2015 that over 620 million children worldwide had untreated ECC, with 1- to 4-year-old children being the most affected [5]. In children under the age of 3 and children aged 3–6, the mean prevalence of ECC was found to be 23.8% and 57.3%, respectively, according to a comprehensive analysis of 193 United Nations published data from 2007 to 2017 [6]. According to the Fourth National Oral Health Epidemiology Survey in the Mainland of China, 50.8%/71.9% of Chinese children aged 3/5 had ECC in 2015, which was higher than ten years ago [7]. Unfortunately, ECC is frequently left untreated, indicating a concerning trend [8]. Given the high prevalence of ECC and the low rates of treatment in China, the need for effective ECC prevention and early treatment is critical.

A high caries burden can have a significant negative impact on a child’s quality of life, causing early pain experiences, reduced food intake, delayed speech development, and diminished self-confidence [9]. It is worth noting that ECC can be greatly reduced by adopting appropriate oral health behaviors and maintaining healthy nutritional and dietary habits [10]. Early and regular dental visits can help prevent the occurrence and progression of dental caries in very young children, potentially resulting in fewer subsequent treatment visits and lower treatment costs [11]. Parents generally regard dentists as knowledgeable about preventive measures, early detection of medical problems, and scientific infant care. Although dentists typically see infants after caries damage has already occurred, they are in a unique position to conduct early examinations, detection, and prevention [2]. Children who have had ECC are at a higher risk of developing subsequent caries than children who have not had ECC. In such cases, preventive measures such as more frequent professional visits for dental examination and restorative care, reinforcement of tooth brushing with fluoride toothpaste, and dietary counseling are required [12].

It is critical for dentists to understand the clinical signs and symptoms of ECC, as well as conduct caries risk assessments and provide preventive guidance for parents. Their cognition and behavior reflect a professional understanding of the importance of promoting children’s oral health. As a result, dental students and residents are thought to have good oral health awareness because they will be responsible for public oral health promotion as the primary providers in the future. Their increased awareness contributes significantly to the prevention of ECC and the preservation of children’s oral health. However, no data on ECC-related knowledge and behavior among dental undergraduates and residents in China are available. Therefore, this study aimed to evaluate the knowledge, attitude, and clinical practice on ECC among dental undergraduates and residents in China, and to provide data for the improvement of future dental education.

## Methods

### Study design and procedures

The survey (approved by the Ethics Committee of the School & Hospital of Stomatology, Wuhan University (2023B12)) was carried out in April–May 2023 among dental undergraduates and residents at the School of Stomatology, Wuhan University, using a structured questionnaire. Participants included both undergraduates (4th and 5th years) and residents (1st, 2nd, and 3rd years). All clinical dental students (4th–5th year) and residents in the 2020–2023 academic year were invited to participate in the study. Each participant was instructed on how to complete the questionnaire and was made aware of the purpose of this survey.

Public discussion groups (WeChat internet platform, Tencent, Shenzhen, China) and Wenjuanxing (Questionnaire collection application, Ranxing Technology, Changsha, China) were employed to distribute the questionnaire using electronic form. A presurvey was conducted to ensure that the questions were understandable before distributing the questionnaire. The questionnaire was distributed to study participants via phone invitation, and informed consent was obtained from all individuals at the start of the questionnaire. The questionnaire used in this survey contained 23 items divided into four sections. At first, participants were asked to provide basic information such as their academic levels. In the first section, participants’ ECC awareness and learning were assessed using six questions (shown in Table 1), such as “Have you ever heard of ECC?” and “Have you received any ECC-related instruction or training?” In the second section, participants’ ECC-related knowledge was assessed using five questions (shown in Table 2), such as “ECC occurs only in bottle-fed infants and children” and “What is the definition of ECC?” In the third section, participants’ ECC-related attitudes were assessed using seven questions (shown in Table 3), such as “Children under the age of 3 can use fluoride toothpaste” and “What is the appropriate age for a child’s first visit to the dentist?” In the fourth section, participants’ ECC-related practice was assessed using four questions (shown in Table 4), such as “Have you ever seen an ECC clinical case?” and “How frequently do you give parents advice on their children’s oral health?” Some of the questions were multiple choice. All participants were notified again as a reminder one week after the questionnaire was delivered. All questionnaires were accomplished anonymously and voluntarily.

**Table 1:** Awareness and learning levels of ECC among dental undergraduates and residents (*N* = 422)

	Responding, n (%)					Total, n (%)	*p* Value by Chi-square test
	Undergraduates		Residents				
	4th-year	5th-year	1st-year	2nd-year	3rd-year		
Have you ever heard of ECC?							
Yes	37 (60.7)	37 (68.5)	77 (84.6)	90 (81.8)	85 (80.2)	326 (77.3)	0.002
No	24 (39.3)	17 (31.5)	14 (15.3)	20 (18.2)	21 (19.8)	96 (22.8)	
Are you familiar with ECC?							
Yes	13 (21.3)	14 (25.9)	26 (28.6)	32 (29.1)	31 (29.3)	116 (27.5)	0.810
No	48 (78.7)	40 (74.1)	65 (71.4)	78 (70.9)	75 (70.8)	306 (72.5)	
Have you received any ECC instruction or training?							
Yes	11 (18.0)	10 (18.5)	19 (20.9)	26 (23.6)	16 (15.1)	82 (19.4)	0.602
No	50 (82.0)	44 (81.5)	72 (79.1)	84 (76.4)	90 (84.9)	340 (80.6)	
Do you want to learn about ECC more systematically?							
Yes	58 (95.1)	50 (92.6)	86 (94.5)	100 (90.9)	102 (96.2)	396 (93.8)	0.548
No	3 (4.9)	4 (7.4)	5 (5.5)	10 (9.1)	4 (3.8)	26 (6.2)	
What are your main ways to learn oral knowledge?							
Textbooks	52 (85.3)	48 (88.9)	59 (64.8)	71 (64.6)	71 (67.0)	301 (71.3)	0.001
Lectures	4 (6.6)	2 (3.7)	15 (16.5)	14 (12.7)	14 (13.2)	49 (11.6)	
Journals	2 (3.3)	2 (3.7)	11 (12.1)	12 (10.9)	19 (17.9)	46 (10.9)	
Internet	3 (4.9)	2 (3.7)	6 (6.6)	13 (11.8)	2 (1.9)	26 (6.2)	
Which part do you want to study in ECC? (multiple choice)							
Etiology	31 (50.8)	25 (46.3)	46 (50.6)	55 (50.0)	57 (53.8)	214 (50.7)	0.933
Epidemiology	31 (50.8)	23 (42.6)	41 (45.0)	42 (38.2)	40 (37.7)	177 (41.9)	0.440
Clinical manifestation	49 (80.3)	42 (77.8)	77 (84.6)	90 (81.8)	81 (76.4)	339 (80.3)	0.651
Therapy	58 (95.1)	51 (94.4)	89 (97.8)	106 (96.4)	100 (94.3)	404 (95.7)	0.764
Prevention	51 (83.6)	48 (88.9)	82 (90.1)	97 (88.2)	92 (86.8)	370 (87.7)	0.807

**Table 2:** Assessment of ECC-related knowledge level of dental undergraduates and residents (*N* = 422)

	Responding correctly, n (%)					Total, n (%)	*p* Value by Chi-square test
	Undergraduates		Residents				
	4th-year	5th-year	1st-year	2nd-year	3rd-year		
The caries prevalence rate of 5-year-old children’s primary teeth in China in recent years							
	6 (9.8)	8 (14.8)	16 (17.6)	19 (17.3)	11 (10.4)	60 (14.2)	0.257
Caries-causing bacteria from the mother’s or guardian’s mouth can colonize the child’s mouth							
	49 (80.3)	41 (75.9)	75 (82.4)	92 (83.6)	92 (86.8)	349 (82.7)	0.748
ECC occurs only in bottle-fed infants and children							
	38 (62.3)	36 (66.7)	67 (73.6)	78 (70.9)	83 (78.3)	302 (71.6)	0.433
The use of fluoride protective paint can effectively prevent and reduce ECC							
	57 (93.4)	52 (96.3)	85 (93.4)	100 (90.9)	100 (94.3)	394 (93.4)	0.520
What is the definition of ECC?							
	31 (50.8)	24 (44.4)	54 (59.3)	57 (51.8)	62 (58.5)	228 (54.0)	0.572

**Table 3:** Assessment of ECC-related attitudes of dental undergraduates and residents (*N* = 422)

	Responding ‘’agree’’, n (%)					Total, n (%)	*p* Value by Chi-square test
	Undergraduates		Residents				
	4th-year	5th-year	1st-year	2nd-year	3rd-year		
Children under the age of 3 can use fluoride toothpaste							
	16 (26.2)	17 (31.5)	54 (59.3)	40 (36.5)	51 (48.1)	178 (42.2)	0.001
Children under the age of 2 should avoid added sugars in their diets							
	29 (47.5)	27 (50.0)	61 (67.0)	58 (52.7)	63 (59.4)	238 (56.4)	0.015
ECC can affect children’s overall health							
	34 (55.7)	33 (61.1)	71 (78.0)	88 (80.0)	86 (81.1)	312 (73.9)	0.000
The frequency and effectiveness of brushing in children can affect the occurrence of ECC							
	54 (88.5)	47 (87.0)	88 (96.7)	104 (94.6)	100 (94.3)	393 (93.1)	0.175
Regular follow-up would be beneficial for controlling ECC							
	59 (96.7)	51 (94.4)	89 (97.8)	107 (97.3)	103 (97.2)	409 (96.9)	0.585
	**Responding, n (%)**					**Total, n (%)**	***p*** **Value by Chi-square test**
	**Undergraduates**		**Residents**				
	**4th-year**	**5th-year**	**1st-year**	**2nd-year**	**3rd-year**		
What is the appropriate age for a child’s first visit to the dentist?							
First primary tooth eruption	30 (49.2)	32 (59.3)	68 (74.7)	83 (75.5)	86 (81.1)	299 (70.9)	0.000
1-year-old	24 (39.3)	8 (14.8)	15 (16.5)	13 (11.8)	8 (7.6)	68 (16.1)	
3-year-old	1 (1.6)	1 (1.9)	5 (5.5)	11 (10.0)	7 (6.6)	25 (5.9)	
Not sure	6 (9.8)	13 (24.1)	3 (3.3)	3 (2.7)	5 (4.7)	30 (7.1)	
When should a child begin brushing their teeth?							
1-year-old	22 (36.1)	17 (31.5)	17 (18.7)	20 (18.2)	17 (16.0)	93 (22.0)	0.105
First primary tooth eruption	33 (54.1)	33 (61.1)	70 (76.9)	81 (73.6)	82 (77.4)	299 (70.9)	
3-year-old	1 (1.6)	0 (0.0)	0 (0.0)	4 (3.6)	2 (1.9)	7 (1.7)	
Preschool	2 (3.3)	0 (0.0)	2 (2.2)	1 (0.9)	0 (0.0)	5 (1.2)	
First permanent tooth eruption	2 (3.3)	3 (5.6)	1 (1.1)	2 (1.8)	3 (2.8)	11 (2.6)	
Not sure	1 (1.6)	1 (1.9)	1 (1.1)	2 (1.8)	2 (1.9)	7 (1.7)	

**Table 4:** Assessment of ECC-related practice of dental undergraduates and residents (*N* = 422)

	Responding, n (%)					Total, n (%)	*p* Value by Chi-square test
	Undergraduates		Residents				
	4th-year	5th-year	1st-year	2nd-year	3rd-year		
Have you ever seen an ECC clinical case?							
Yes	20 (32.8)	27 (50.0)	41 (45.0)	59 (53.6)	65 (61.3)	212 (50.2)	0.003
No	38 (62.3)	24 (44.4)	38 (41.8)	45 (40.9)	30 (28.3)	175 (41.5)	
Not sure	3 (4.9)	3 (5.6)	12 (13.2)	6 (5.5)	11 (10.4)	35 (8.3)	
Have you ever examined and diagnosed an ECC case?							
Yes	7 (11.5)	5 (9.26)	21 (23.1)	28 (25.5)	22 (20.8)	83 (19.7)	0.052
No	54 (88.5)	49 (90.7)	70 (76.9)	82 (74.6)	84 (79.3)	339 (80.3)	
How frequently do you treat caries for children in the clinic?							
Daily	1 (1.6)	4 (7.4)	4 (4.4)	5 (4.6)	2 (1.9)	16 (3.8)	0.000
Weekly	5 (8.2)	7 (13.0)	19 (20.9)	28 (25.6)	34 (32.1)	93 (22.0)	
Month	7 (11.5)	8 (14.8)	29 (31.9)	36 (32.7)	28 (26.4)	108 (25.6)	
Yearly	48 (78.7)	35 (64.8)	39 (42.9)	41 (37.3)	42 (39.6)	205 (48.6)	
How frequently do you give parents advice on their children’s oral health?							
Always	23 (37.7)	22 (40.7)	40 (44.0)	60 (54.6)	52 (49.1)	197 (46.7)	0.159
Sometimes	21 (34.4)	13 (24.1)	23 (25.3)	20 (18.2)	28 (26.4)	105 (24.9)	
Occasionally	11 (18.0)	16 (29.6)	21 (23.1)	26 (23.6)	15 (14.2)	89 (21.1)	
Never	6 (9.8)	3 (5.6)	7 (7.7)	4 (3.6)	11 (10.4)	31 (7.4)	

### Data analysis

All data collected were employed for statistical analysis with SPSS software v. 26.0 (IBM, Armonk, USA). Tables of distribution and frequency were used to present the descriptive statistical analysis. Categorical data were expressed as percentages and then analyzed. The distributions were compared using Pearson’s Chi-square test. If necessary, the Bonferroni test, Z-test, or Fisher’s exact test were applied. A significant difference was defined at 0.05.

## Results

### Participants

This survey distributed 598 questionnaires, of which 422 were returned. The recovery rate for questionnaires was calculated to be 70.6% (Table 5). According to the percentage of all returned questionnaires, 14.4% were 4th‑year undergraduates, 12.8% were 5th‑year undergraduates, 21.6% were 1st‑year residents, 26.1% were 2nd‑year residents, and 25.1% were 3rd‑year residents.

**Table 5:** Demographic information of participants involved in the questionnaires (*N* = 422)

Grade	Target number (n)	Recovery number (n)	Recovery rate (%)
4th-year undergraduates	80	61	76.3
5th-year undergraduates	72	54	67.5
1st-year residents	143	91	63.6
2nd-year residents	146	110	75.3
3rd-year residents	157	106	67.5
Total	598	422	70.6

### ECC-related awareness and knowledge

ECC-related awareness and learning levels in Table 1 show that 77.3% of participants had heard of ECC, with undergraduates reporting a significantly lower rate than residents (*p* < 0.05). However, only 27.5% of participants considered themselves familiar with ECC, and there was no significant difference between undergraduates and residents (*p* > 0.05). For the majority of participants (71.3%), textbooks were their primary source of ECC information. Moreover, undergraduates preferred textbooks over residents (*p* < 0.05), while 3rd‑year residents gained more ECC knowledge from journals than other grades (*p* < 0.05). Surprisingly, only 19.4% of participants received ECC instruction or training. In terms of specific areas of interest, participants were more interested in the clinical manifestation (80.3%), prevention (87.7%), and therapy (95.7%) of ECC. Approximately 50.7% and 41.9% of participants expressed a desire to learn about etiology and epidemiology, respectively. Furthermore, 93.8% of participants wished to learn about ECC more systematically.

Table 2 summarizes ECC-related knowledge, including its definition, etiology, epidemiology, and prevention strategy. Nearly half of the participants (54.0%) correctly defined ECC, which is considered required to master in our undergraduate syllabus, and no significant difference was noted between undergraduates and residents (*p* > 0.05). For the epidemiology of ECC in China reported in recent years, only 14.2% of participants provided the correct answer (with no significant difference between undergraduates and residents, (*p* > 0.05), indicating a significant lack of attention to ECC. The majority of participants (82.7%/71.6%) agreed that caries-causing bacteria from the mother’s or guardian’s mouth can colonize the child’s mouth and that ECC does not occur only in bottle-fed infants and children, respectively. When it comes to ECC prevention, almost all participants (93.4%) approved that using fluoride can effectively prevent ECC.

### ECC-related attitudes

As shown in Table 3, only 42.2% of participants agreed that children under the age of 3 can use fluoride toothpaste. In terms of sugar intake, a higher percentage of residents (59.7%) agreed that children under the age of 2 should avoid added sugars in their diets than undergraduates (48.8%) (*p* < 0.05). Most participants (94.1%) agreed that the frequency and effectiveness of brushing in children can affect the occurrence of ECC. Residents (79.8%) had higher risk awareness of ECC on children’s overall health than undergraduates (58.3%) (*p* < 0.05).

When it comes to the timing of tooth brushing for children, more residents (75.9%) than undergraduates (57.4%) believed it is appropriate to begin brushing after the first primary tooth eruption (*p* < 0.05). Few participants (2.6%) held that children should begin brushing after permanent tooth eruption. Furthermore, most residents (77.2%) believed that the age of a child’s first visit to the dentist should be at 6 months, which differed significantly from the views of undergraduates (53.9%) (*p* < 0.05).

### ECC-related clinical practice

The clinical practice results for ECC are summarized in Table 4. Approximately half of the participants had encountered ECC cases in clinical practice, with undergraduates reporting these experiences at a significantly lower rate (40.9%) than residents (53.7%) (*p* < 0.05). Moreover, a higher proportion of residents (23.1%) had ever examined and diagnosed for ECC, which is significantly higher than undergraduates (10.4%) (*p* < 0.05). Nearly half of the participants (48.6%) reported that they treated ECC in the clinic on a yearly basis, with only a few participants experiencing it on a daily (3.8%), weekly (22.0%), or monthly (25.6%) basis. Only 46.7% of participants always provided oral health advice to parents for their children, and 7.4% never did.

## Discussion

ECC has been a worldwide public concern due to its high prevalence rate and low treatment rate, as well as but its significant impact on the life quality and well-being of children and their families. Children with ECC may suffer from a variety of impairments in various aspects of their lives [13]. In this study, the majority of participants had heard about ECC but were unfamiliar with it, implying that ECC-related instruction or training is insufficient in the current situation. Most dental undergraduates and residents learn ECC knowledge primarily from textbooks, especially undergraduates. The findings of the source of theoretical knowledge study are consistent with those of a previous study [14]. Textbooks are typically classical books on one single professional field. However, pediatric dentistry textbooks used in dental education include only a limited portion of ECC, which cannot satisfy the comprehensive learning and understanding of ECC [15]. In addition to textbooks, undergraduates showed a considerable decrease in the proportion of learning oral knowledge through lectures, journals, and the internet when compared to residents. This could be related to the fact that undergraduates in China begin clinical practice in their final year without any theoretical study [14], and their rotations in some departments are rushed. The transition from dental student to independent dentist will take place during residency. Residents are required to attend theoretical training lectures related to clinical practice on a regular basis at the School of Stomatology, Wuhan University. Previous surveys also found such a lack of knowledge mastery in dental education [16, 17]. Our findings indicated that the activity and problem-based learning of undergraduates and residents should be encouraged and enhanced during clinical practice education.

Despite the fact that ECC is a highly prevalent disease among children and is frequently encountered in dental clinics and hospitals, an extremely low percentage of participants were aware of the prevalence of caries among 5-year-old children in China in recent years, indicating an inadequate active and passive updating of professional knowledge. The non-ideal outcome is also reflected in the low level of reporting the correct definition of ECC. Nonetheless, a large majority of participants are eager to learn about ECC more systematically, particularly in terms of clinical manifestation, therapy, and prevention, implying their enthusiasm for learning ECC-related information. It is worth noting that not only clinical skill training but also ECC theoretical knowledge teaching during dental education should be prioritized and consolidated.

A variety of antimicrobial agents have been employed to prevent dental caries [18–20], with fluoride toothpaste being the most recommended in recent decades [21, 22]. In both optimally fluoridated and fluoride-deficient communities, it is currently rational practice for all children to brush their teeth twice a day with fluoride toothpaste to lower the risk of ECC [23, 24]. The interaction with oral plaque biofilm inhibited the growth of *Streptococcus mutans* and *Porphyromonas gingivalis* after fluoride toothpaste treatment [25]. However, less than half of the participants believed that children under the age of 3 should brush their teeth with fluoride toothpaste, denoting that they were unfamiliar with the use of fluoride toothpaste for children. The findings were consistent with a population-based study that found only 31.3% of dentists had a good understanding of the benefits and risks of fluoride toothpaste in children under the age of [26]. The use of fluoride toothpaste at a concentration of ≥ 1000 ppm on a regular basis helps to reduce caries progression [27]. As a result, more continuing dental education is demanded to improve the knowledge mastery of the use of fluoride toothpaste among undergraduates and residents. Furthermore, the majority of participants agreed that the frequency and effectiveness of brushing in children can affect the occurrence of ECC. It has been demonstrated that less than twice-daily tooth brushing and difficulties with brushing procedures during the preschool years were significant predictors of caries prevalence at the age of 5 [28]. These findings suggested that the knowledge and preventive measures are insufficient, and that more theoretical education is required.

The level of knowledge, attitude, and practice in ECC cases has a direct impact on dentists’ ability to provide professional services for early childhood. The American Academy of Pediatric Dentistry (AAPD) recommends a first visit within 6 months of the eruption of the first primary tooth, or around 1 year of age [21]. Several studies suggest that the occurrence and development of ECC can be reduced through early and regular dental visits, particularly within the first year of life [1, 2]. In this study, most participants gave the correct timing for children’s first dental visit and teeth cleaning. Moreover, nearly all of the participants believed that regular follow-up examinations every 6 months aid in the reduction of ECC. This evidence demonstrated that the participants knows were well informed about the importance of early dental visits in ECC prevention. In comparison to undergraduates, residents exhibited greater proficiency in the prevention of ECC, as indicated by Table 3. Essentially, the duration of clinical practice at the School of Stomatology, Wuhan University, was one week for 5th-year undergraduates and three months for residents [14]. Over the course of the extended clinical practice, residents are more likely to gain additional knowledge regarding the etiology and preventive measures of ECC. Consequently, in order to improve undergraduates’ understanding of ECC prevention, it is crucial to give greater emphasis on ECC education and to extend their clinical practice time.

Recent research has consistently found that a lack of access to dental services is a critical risk factor that negatively affects preschool children’s oral health [29, 30]. Dentists play an important role in improving the oral health of children by assessing the risk of ECC and providing primary prevention and appropriate therapeutic services. Dental caries is a common reason for parents or other guardians to refer their children to a dentist. The majority of participants agreed that pediatric dentists are crucial for preventing ECC. Pediatric dentists are on the frontlines of identifying and referring patients with caries or high-risk carious lesions. According to the results in Table 4, residents had more clinical experience dealing with ECC than undergraduates, with 4th‑year undergraduates having the lowest proportion. Children with ECC are more commonly encountered by dental residents in their daily work at the Hospital of Stomatology, Wuhan University. However, it should be mentioned that in the majority of Chinese universities and colleges, the duration of clinical practice varies for undergraduates and residents. Regarding Wuhan University, the clinical practice lasts for several days (less than one week) for 4th‑year undergraduates, one week for 5th‑year undergraduates, and three months for residents [14]. The undergraduates are not exposed to adequate clinical practice because they spend a brief period of time at the pediatric dentistry department before becoming familiar with the systematic treatment for ECC. These reasons explain why residents had more experience than undergraduates, with 4th‑year undergraduates having the least.

It has been reported that in many countries, more than 90% of ECC patients go untreated [31]. In China, only about 3.1% of children with caries receive treatment [7]. It is primarily because ECC treatment is generally costly and time-consuming, requiring experienced pediatric dentists. Our results revealed that only a few participants had ever treated children with ECC. Dental students and residents are frequently at a loss when confronted with children and parents in clinical practice, and they do not know how to soothe children’s negative emotions. As a result, dental care is frequently unavailable. Furthermore, the low treatment for ECC may be due to the students’ inaccuracy in indication of ECC treatment. The Chinese Dental Association published guidelines for the prevention and treatment of caries in infants and children in 2021 [32], covering a comprehensive approach to childhood caries management. This is expected to contribute to the improvement of the guidance on ECC-related knowledge and prevention.

Almost half of the participants reported that they do not always provide oral health advice to parents for their children, which may be related to an inaccurate understanding of ECC. Dentists could offer target-specific preventive measures to effectively combat the disease by identifying “high-caries-risk” individuals and associated risk factors [33]. However, it is noteworthy that systematic ECC training is relatively lacking in China. In general, ECC education makes up only a small portion of pediatric dentistry education (roughly 5%~10% of final exam scores) and is mainly intended to assess students’ grasp of the definition and clinical manifestation of ECC. During the theoretical teaching and practical training, there was little emphasis placed on the local and global epidemiological situation, etiology, early prevention measures, and treatment approaches [34]. Undergraduates had far less experience with pediatric dentistry than residents did, and their attitudes were primarily influenced by their knowledge and experience. Therefore, compared to residents, the knowledge, attitude, and clinical practice of undergraduates regarding ECC were less optimal. Concerning the role of dentists in community health, it is critical to focus more on professional behavior education for undergraduates and residents. The sample size in this study was sufficient for data analysis, but the results should be interpreted carefully because the current findings are limited to the city of Wuhan. Future studies with a broad inclusion of dental students at various levels and schools are recommended, as they would be more representative of the Chinese dental student population.

## Conclusion

There were some knowledge gaps regarding ECC among dental undergraduates and residents in China, particularly with regard to the epidemiology and clinical manifestation. They obtained ECC knowledge in a straightforward manner, and there may be a lack of ECC continuing education in clinical practice. However, the majority of dental undergraduates and residents are eager to learn more about ECC. As a result, it is recommended that more comprehensive, systematic education on ECC should be provided during their clinical practice. Paediatric dentists and dental educators should work together to facilitate the reform of dental education that will enhance the promotion of children’s oral health.

## Data Availability

The data that support the findings of this study are available from the corresponding author upon reasonable request.
